# Supplementary material for: Pyruvate Dehydrogenase Kinase 1 inhibition mediated oxidative phosphorylation enhancement in cartilage promotes osteoarthritis progression
Source: BMC Musculoskelet Disord. 2023 Jul 20;24:597. doi: 10.1186/s12891-023-06585-6 (PMC10357736; doi:10.1186/s12891-023-06585-6)
Supplement: Supplementary file 1 — Additional file 1. [file 12891_2023_6585_MOESM1_ESM.zip › MF.Gsea.1653627316242/GOMF_PROTEIN_TYROSINE_PHOSPHATASE_ACTIVITY.html]

Details for gene set GOMF\_PROTEIN\_TYROSINE\_PHOSPHATASE\_ACTIVITY[GSEA]

|  || Dataset | 55457\_collapsed\_to\_symbols.cls.cls #OA\_versus\_normal.cls.cls #OA\_versus\_normal\_repos |
| Phenotype | cls.cls#OA\_versus\_normal\_repos |
| Upregulated in class | normal |
| GeneSet | GOMF\_PROTEIN\_TYROSINE\_PHOSPHATASE\_ACTIVITY |
| Enrichment Score (ES) | -0.37305248 |
| Normalized Enrichment Score (NES) | -1.5688034 |
| Nominal p-value | 0.014173228 |
| FDR q-value | 0.15603316 |
| FWER p-Value | 0.999 |
Table: GSEA Results Summary

  

Fig 1: Enrichment plot: GOMF\_PROTEIN\_TYROSINE\_PHOSPHATASE\_ACTIVITY      
 Profile of the Running ES Score & Positions of GeneSet Members on the Rank Ordered List

  

| SYMBOL | TITLE | RANK IN GENE LIST | RANK METRIC SCORE | RUNNING ES | CORE ENRICHMENT || 1 | EYA2 | EYA transcriptional coactivator and phosphatase 2 [Source:HGNC Symbol;Acc:HGNC:3520] | 219 | 0.807 | 0.0113 | No |
| 2 | PTPRO | protein tyrosine phosphatase receptor type O [Source:HGNC Symbol;Acc:HGNC:9678] | 364 | 0.719 | 0.0253 | No |
| 3 | PTPN22 | protein tyrosine phosphatase non-receptor type 22 [Source:HGNC Symbol;Acc:HGNC:9652] | 578 | 0.630 | 0.0308 | No |
| 4 | DUSP22 | dual specificity phosphatase 22 [Source:HGNC Symbol;Acc:HGNC:16077] | 998 | 0.518 | 0.0163 | No |
| 5 | PTPN9 | protein tyrosine phosphatase non-receptor type 9 [Source:HGNC Symbol;Acc:HGNC:9661] | 1119 | 0.492 | 0.0242 | No |
| 6 | PTPRR | protein tyrosine phosphatase receptor type R [Source:HGNC Symbol;Acc:HGNC:9680] | 1352 | 0.450 | 0.0219 | No |
| 7 | CDKN3 | cyclin dependent kinase inhibitor 3 [Source:HGNC Symbol;Acc:HGNC:1791] | 1422 | 0.440 | 0.0319 | No |
| 8 | PTPRA | protein tyrosine phosphatase receptor type A [Source:HGNC Symbol;Acc:HGNC:9664] | 1698 | 0.399 | 0.0245 | No |
| 9 | DUSP12 | dual specificity phosphatase 12 [Source:HGNC Symbol;Acc:HGNC:3067] | 1802 | 0.384 | 0.0299 | No |
| 10 | PTPN7 | protein tyrosine phosphatase non-receptor type 7 [Source:HGNC Symbol;Acc:HGNC:9659] | 1864 | 0.377 | 0.0384 | No |
| 11 | DUSP14 | dual specificity phosphatase 14 [Source:HGNC Symbol;Acc:HGNC:17007] | 1921 | 0.370 | 0.0471 | No |
| 12 | PTPRN2 | protein tyrosine phosphatase receptor type N2 [Source:HGNC Symbol;Acc:HGNC:9677] | 1965 | 0.365 | 0.0565 | No |
| 13 | PTPN18 | protein tyrosine phosphatase non-receptor type 18 [Source:HGNC Symbol;Acc:HGNC:9649] | 2715 | 0.278 | 0.0079 | No |
| 14 | PTPRD | protein tyrosine phosphatase receptor type D [Source:HGNC Symbol;Acc:HGNC:9668] | 3094 | 0.240 | -0.0132 | No |
| 15 | DUSP11 | dual specificity phosphatase 11 [Source:HGNC Symbol;Acc:HGNC:3066] | 3135 | 0.237 | -0.0080 | No |
| 16 | PTPRT | protein tyrosine phosphatase receptor type T [Source:HGNC Symbol;Acc:HGNC:9682] | 3157 | 0.234 | -0.0014 | No |
| 17 | PTPN6 | protein tyrosine phosphatase non-receptor type 6 [Source:HGNC Symbol;Acc:HGNC:9658] | 3434 | 0.208 | -0.0157 | No |
| 18 | DNAJC6 | DnaJ heat shock protein family (Hsp40) member C6 [Source:HGNC Symbol;Acc:HGNC:15469] | 3665 | 0.189 | -0.0270 | No |
| 19 | SSH3 | slingshot protein phosphatase 3 [Source:HGNC Symbol;Acc:HGNC:30581] | 3707 | 0.185 | -0.0237 | No |
| 20 | PPP2CA | protein phosphatase 2 catalytic subunit alpha [Source:HGNC Symbol;Acc:HGNC:9299] | 4146 | 0.143 | -0.0529 | No |
| 21 | EYA3 | EYA transcriptional coactivator and phosphatase 3 [Source:HGNC Symbol;Acc:HGNC:3521] | 4258 | 0.132 | -0.0569 | No |
| 22 | PTPN3 | protein tyrosine phosphatase non-receptor type 3 [Source:HGNC Symbol;Acc:HGNC:9655] | 4312 | 0.128 | -0.0565 | No |
| 23 | PTPN13 | protein tyrosine phosphatase non-receptor type 13 [Source:HGNC Symbol;Acc:HGNC:9646] | 4342 | 0.125 | -0.0544 | No |
| 24 | MTMR7 | myotubularin related protein 7 [Source:HGNC Symbol;Acc:HGNC:7454] | 4432 | 0.119 | -0.0572 | No |
| 25 | RNGTT | RNA guanylyltransferase and 5'-phosphatase [Source:HGNC Symbol;Acc:HGNC:10073] | 4737 | 0.092 | -0.0777 | No |
| 26 | ACP1 | acid phosphatase 1 [Source:HGNC Symbol;Acc:HGNC:122] | 4772 | 0.089 | -0.0772 | No |
| 27 | PTPRF | protein tyrosine phosphatase receptor type F [Source:HGNC Symbol;Acc:HGNC:9670] | 4839 | 0.084 | -0.0794 | No |
| 28 | DUSP3 | dual specificity phosphatase 3 [Source:HGNC Symbol;Acc:HGNC:3069] | 4857 | 0.083 | -0.0778 | No |
| 29 | CDC14B | cell division cycle 14B [Source:HGNC Symbol;Acc:HGNC:1719] | 5099 | 0.064 | -0.0944 | No |
| 30 | PTPN20 | protein tyrosine phosphatase non-receptor type 20 [Source:HGNC Symbol;Acc:HGNC:23423] | 5240 | 0.053 | -0.1034 | No |
| 31 | PTPN12 | protein tyrosine phosphatase non-receptor type 12 [Source:HGNC Symbol;Acc:HGNC:9645] | 5310 | 0.047 | -0.1072 | No |
| 32 | MTMR14 | myotubularin related protein 14 [Source:HGNC Symbol;Acc:HGNC:26190] | 5458 | 0.037 | -0.1174 | No |
| 33 | PTPN4 | protein tyrosine phosphatase non-receptor type 4 [Source:HGNC Symbol;Acc:HGNC:9656] | 5563 | 0.027 | -0.1245 | No |
| 34 | CDC25A | cell division cycle 25A [Source:HGNC Symbol;Acc:HGNC:1725] | 5666 | 0.018 | -0.1319 | No |
| 35 | MTM1 | myotubularin 1 [Source:HGNC Symbol;Acc:HGNC:7448] | 5768 | 0.010 | -0.1394 | No |
| 36 | DUSP13 | dual specificity phosphatase 13 [Source:HGNC Symbol;Acc:HGNC:19681] | 6110 | -0.013 | -0.1655 | No |
| 37 | HACD2 | 3-hydroxyacyl-CoA dehydratase 2 [Source:HGNC Symbol;Acc:HGNC:9640] | 6162 | -0.017 | -0.1689 | No |
| 38 | MTMR2 | myotubularin related protein 2 [Source:HGNC Symbol;Acc:HGNC:7450] | 6189 | -0.019 | -0.1702 | No |
| 39 | ACP3 | acid phosphatase 3 [Source:HGNC Symbol;Acc:HGNC:125] | 6271 | -0.025 | -0.1757 | No |
| 40 | PTPRK | protein tyrosine phosphatase receptor type K [Source:HGNC Symbol;Acc:HGNC:9674] | 6463 | -0.040 | -0.1892 | No |
| 41 | MTMR4 | myotubularin related protein 4 [Source:HGNC Symbol;Acc:HGNC:7452] | 6899 | -0.072 | -0.2206 | No |
| 42 | DUSP7 | dual specificity phosphatase 7 [Source:HGNC Symbol;Acc:HGNC:3073] | 6966 | -0.077 | -0.2230 | No |
| 43 | PTPRZ1 | protein tyrosine phosphatase receptor type Z1 [Source:HGNC Symbol;Acc:HGNC:9685] | 7123 | -0.090 | -0.2321 | No |
| 44 | PGP | phosphoglycolate phosphatase [Source:HGNC Symbol;Acc:HGNC:8909] | 7203 | -0.097 | -0.2348 | No |
| 45 | PTPRC | protein tyrosine phosphatase receptor type C [Source:HGNC Symbol;Acc:HGNC:9666] | 7744 | -0.137 | -0.2721 | No |
| 46 | DUSP10 | dual specificity phosphatase 10 [Source:HGNC Symbol;Acc:HGNC:3065] | 7775 | -0.140 | -0.2696 | No |
| 47 | PTP4A3 | protein tyrosine phosphatase 4A3 [Source:HGNC Symbol;Acc:HGNC:9636] | 7984 | -0.154 | -0.2804 | No |
| 48 | PTPRB | protein tyrosine phosphatase receptor type B [Source:HGNC Symbol;Acc:HGNC:9665] | 8044 | -0.158 | -0.2794 | No |
| 49 | MTMR1 | myotubularin related protein 1 [Source:HGNC Symbol;Acc:HGNC:7449] | 8102 | -0.163 | -0.2782 | No |
| 50 | DUSP21 | dual specificity phosphatase 21 [Source:HGNC Symbol;Acc:HGNC:20476] | 8323 | -0.180 | -0.2890 | No |
| 51 | DUSP26 | dual specificity phosphatase 26 [Source:HGNC Symbol;Acc:HGNC:28161] | 8424 | -0.187 | -0.2902 | No |
| 52 | TIMM50 | translocase of inner mitochondrial membrane 50 [Source:HGNC Symbol;Acc:HGNC:23656] | 8731 | -0.211 | -0.3067 | No |
| 53 | PTP4A2 | protein tyrosine phosphatase 4A2 [Source:HGNC Symbol;Acc:HGNC:9635] | 8806 | -0.217 | -0.3049 | No |
| 54 | PTPN1 | protein tyrosine phosphatase non-receptor type 1 [Source:HGNC Symbol;Acc:HGNC:9642] | 8829 | -0.220 | -0.2988 | No |
| 55 | PTP4A1 | protein tyrosine phosphatase 4A1 [Source:HGNC Symbol;Acc:HGNC:9634] | 8874 | -0.223 | -0.2945 | No |
| 56 | PTPRJ | protein tyrosine phosphatase receptor type J [Source:HGNC Symbol;Acc:HGNC:9673] | 9463 | -0.270 | -0.3309 | No |
| 57 | CDC25C | cell division cycle 25C [Source:HGNC Symbol;Acc:HGNC:1727] | 9501 | -0.274 | -0.3241 | No |
| 58 | SSH1 | slingshot protein phosphatase 1 [Source:HGNC Symbol;Acc:HGNC:30579] | 9748 | -0.296 | -0.3329 | No |
| 59 | TPTE | transmembrane phosphatase with tensin homology [Source:HGNC Symbol;Acc:HGNC:12023] | 10002 | -0.321 | -0.3414 | No |
| 60 | EYA4 | EYA transcriptional coactivator and phosphatase 4 [Source:HGNC Symbol;Acc:HGNC:3522] | 10068 | -0.328 | -0.3349 | No |
| 61 | CDC25B | cell division cycle 25B [Source:HGNC Symbol;Acc:HGNC:1726] | 10206 | -0.341 | -0.3336 | No |
| 62 | PTPRN | protein tyrosine phosphatase receptor type N [Source:HGNC Symbol;Acc:HGNC:9676] | 10712 | -0.395 | -0.3592 | Yes |
| 63 | MTMR3 | myotubularin related protein 3 [Source:HGNC Symbol;Acc:HGNC:7451] | 10751 | -0.399 | -0.3482 | Yes |
| 64 | EYA1 | EYA transcriptional coactivator and phosphatase 1 [Source:HGNC Symbol;Acc:HGNC:3519] | 10878 | -0.414 | -0.3435 | Yes |
| 65 | MTMR8 | myotubularin related protein 8 [Source:HGNC Symbol;Acc:HGNC:16825] | 10991 | -0.429 | -0.3371 | Yes |
| 66 | PTPRS | protein tyrosine phosphatase receptor type S [Source:HGNC Symbol;Acc:HGNC:9681] | 11017 | -0.432 | -0.3239 | Yes |
| 67 | MTMR6 | myotubularin related protein 6 [Source:HGNC Symbol;Acc:HGNC:7453] | 11085 | -0.442 | -0.3136 | Yes |
| 68 | PTPN11 | protein tyrosine phosphatase non-receptor type 11 [Source:HGNC Symbol;Acc:HGNC:9644] | 11260 | -0.465 | -0.3108 | Yes |
| 69 | PTPN2 | protein tyrosine phosphatase non-receptor type 2 [Source:HGNC Symbol;Acc:HGNC:9650] | 11270 | -0.467 | -0.2951 | Yes |
| 70 | PTPRM | protein tyrosine phosphatase receptor type M [Source:HGNC Symbol;Acc:HGNC:9675] | 11289 | -0.470 | -0.2800 | Yes |
| 71 | DUSP9 | dual specificity phosphatase 9 [Source:HGNC Symbol;Acc:HGNC:3076] | 11486 | -0.500 | -0.2778 | Yes |
| 72 | PTPN21 | protein tyrosine phosphatase non-receptor type 21 [Source:HGNC Symbol;Acc:HGNC:9651] | 11508 | -0.502 | -0.2618 | Yes |
| 73 | DUSP2 | dual specificity phosphatase 2 [Source:HGNC Symbol;Acc:HGNC:3068] | 11768 | -0.544 | -0.2629 | Yes |
| 74 | PTPRE | protein tyrosine phosphatase receptor type E [Source:HGNC Symbol;Acc:HGNC:9669] | 11787 | -0.547 | -0.2451 | Yes |
| 75 | PTPN14 | protein tyrosine phosphatase non-receptor type 14 [Source:HGNC Symbol;Acc:HGNC:9647] | 11797 | -0.548 | -0.2266 | Yes |
| 76 | PTEN | phosphatase and tensin homolog [Source:HGNC Symbol;Acc:HGNC:9588] | 11836 | -0.556 | -0.2100 | Yes |
| 77 | TNS2 | tensin 2 [Source:HGNC Symbol;Acc:HGNC:19737] | 11933 | -0.580 | -0.1971 | Yes |
| 78 | DUSP4 | dual specificity phosphatase 4 [Source:HGNC Symbol;Acc:HGNC:3070] | 12068 | -0.612 | -0.1861 | Yes |
| 79 | DUSP8 | dual specificity phosphatase 8 [Source:HGNC Symbol;Acc:HGNC:3074] | 12303 | -0.678 | -0.1805 | Yes |
| 80 | PTPRH | protein tyrosine phosphatase receptor type H [Source:HGNC Symbol;Acc:HGNC:9672] | 12464 | -0.732 | -0.1673 | Yes |
| 81 | PTPRG | protein tyrosine phosphatase receptor type G [Source:HGNC Symbol;Acc:HGNC:9671] | 12564 | -0.779 | -0.1477 | Yes |
| 82 | DUSP6 | dual specificity phosphatase 6 [Source:HGNC Symbol;Acc:HGNC:3072] | 12644 | -0.821 | -0.1250 | Yes |
| 83 | DUSP5 | dual specificity phosphatase 5 [Source:HGNC Symbol;Acc:HGNC:3071] | 12721 | -0.882 | -0.1000 | Yes |
| 84 | CDC14A | cell division cycle 14A [Source:HGNC Symbol;Acc:HGNC:1718] | 12823 | -1.009 | -0.0724 | Yes |
| 85 | PTPRU | protein tyrosine phosphatase receptor type U [Source:HGNC Symbol;Acc:HGNC:9683] | 12829 | -1.026 | -0.0368 | Yes |
| 86 | DUSP1 | dual specificity phosphatase 1 [Source:HGNC Symbol;Acc:HGNC:3064] | 12884 | -1.202 | 0.0012 | Yes |
Table: GSEA details [plain text format]

  

Fig 2: GOMF\_PROTEIN\_TYROSINE\_PHOSPHATASE\_ACTIVITY      
 Blue-Pink O' Gram in the Space of the Analyzed GeneSet

  

Fig 3: GOMF\_PROTEIN\_TYROSINE\_PHOSPHATASE\_ACTIVITY: Random ES distribution      
 Gene set null distribution of ES for **GOMF\_PROTEIN\_TYROSINE\_PHOSPHATASE\_ACTIVITY**
